# Impact of coccidiostat and phytase supplementation on gut microbiota composition and phytate degradation in broiler chickens

**DOI:** 10.1186/s42523-019-0006-2

**Published:** 2019-06-28

**Authors:** Susanne Künzel, Daniel Borda-Molina, Rebecca Kraft, Vera Sommerfeld, Imke Kühn, Amélia Camarinha-Silva, Markus Rodehutscord

**Affiliations:** 1 Institut für Nutztierwissenschaften, Universität Hohenheim, 70599 Stuttgart, Germany; 2 AB Vista, 64293 Darmstadt, Germany

**Keywords:** 16S rRNA gene, Microbiota, Coccidiostat, Phytase, Phytate, Crude protein, Broiler, Narasin, Nicarbazin

## Abstract

**Background:**

There is good evidence for a substantial endogenous phytase activity originating from the epithelial tissue or the microbiota resident in the digestive tract of broiler chickens. However, ionophore coccidiostats, which are frequently used as feed additives in broiler diets to prevent coccidiosis, might affect the bacterial composition and the abundance of phytase producers in the gastrointestinal tract. The aim of the present study was to investigate whether supplementation of a frequently used mixture of the coccidiostats Narasin and Nicarbazin alone or together with a phytase affects microbiota composition of the digestive tract of broiler chickens, characteristics of phytate breakdown in crop and terminal ileum, and precaecal phosphorus and crude protein digestibility.

**Results:**

Large differences in the microbial composition and diversity were detected between the treatments with and without coccidiostat supplementation. Disappearance of *myo*-inositol 1,2,3,4,5,6-hexakis(dihydrogen phosphate) (InsP_6_) in the digestive tract, precaecal P digestibility, inorganic P in blood serum, and the concentration of inositol phosphate isomers in the crop and ileum digesta were significantly affected by phytase supplementation, but not by coccidiostat supplementation. Crude protein digestibility was increased by coccidiostat supplementation when more phosphate was available. Neither microbial abundance and diversity nor any other trait measured at the end of the experiment was affected by coccidiostat when it was only supplemented from day 1 to 10 of age.

**Conclusions:**

The coccidiostats used herein had large effects on overall microbiota composition of the digestive tract. The coccidiostats did not seem to affect endogenous or exogenous phytase activity up to the terminal ileum of broiler chickens. The effects of phytase on growth, phosphorus digestibility, and *myo*-inositol release were not altered by the presence of the coccidiostats. The effects of phytase and coccidiostats on nutrient digestibility can be of significant relevance for phosphorus and protein-reduced feeding concepts if confirmed in further experiments.

**Electronic supplementary material:**

The online version of this article (10.1186/s42523-019-0006-2) contains supplementary material, which is available to authorized users.

## Background

Phosphorus (P) has several essential effects in birds´ metabolism, hence it is an important element in poultry nutrition. The main ingredients of poultry diets are plant seeds and by-products from seed processing. In these feed ingredients, P is mainly bound as *myo*-inositol 1,2,3,4,5,6-hexakis(dihydrogen phosphate) (InsP_6_) and its salts (phytate), and in this form only partially available to poultry. Recent studies have shown a high potential for degradation of InsP_6_ in the digestive tract (56 – 89 %) when broiler chickens were provided with diets having low P and calcium (Ca) contents and phytate-degrading enzymes (phytase) not added [1–4]. This points towards a substantial endogenous phytase activity originating from the epithelial tissue or the microbiota resident in the digestive tract [5, 6].

A comprehensive P digestibility ring test [7] has shown that P digestibility and InsP_6_ disappearance until the end of the ileum (precaecal) in broilers varied to a large extent between different institutions although the same experimental diets were used by all participants of the ring test and the trial protocol was standardized [8]. The authors speculated that coccidiostats used in the pre-experimental phase in some but not all institutions have contributed to this variation by influencing phytase-producing bacteria. Coccidiostats are a group of specific agents that are approved as feed additives (not meaning a medicinal product for therapy) in the EU [9, 10] for prevention of coccidiosis. Ionophore coccidiostats are known to have an effect on the microbial composition of the gastrointestinal tract [11, 12]. For example, Ludvigsen et al. reported a significant decrease of *Clostridium perfringens* in cecal content of narasin treated broilers [13]. Some microorganisms are known as phytase producers, many of them belonging to *Lactobacillus* species, such as *L. salivarius*, *L. brevis, L. plantarum* and *L. pentosus* [14–17]. *L. salivarius* is often found to be part of the gut microbiota of broiler chickens, specifically in the crop [18, 19]. Supplementation of antimicrobial products to poultry diets might change the proportion of phytase producing bacteria in the microbial community. A decrease of *L. salivarius* has been detected in caecal content of broiler chickens fed with diets supplemented with the antibacterial agent zinc bacitracin [20].

No study investigated effects of coccidiostats on P utilization in non-ruminants to date and only few studies investigated effects of antibacterial products. In broiler chickens and pigs, no significant effect of the antibacterial products tylosin or virginiamycin on precaecal P digestibility was found [21]. In other studies, the antibacterial agents virginiamycin and cyadox increased apparent P digestibility in pigs [22, 23]. The authors are not aware of any studies investigating the effects of in-feed antimicrobials on InsP_6_ degradation in the digestive tract of broiler chickens and related microbiota composition.

Therefore, our objective was to study whether a widely used coccidiostat feed additive which is a mixture of a chemical (Nicarbazin) and an ionophore (Narasin) coccidiostat could affect InsP_6_ disappearance in the digestive tract, precaecal P digestibility, and the microbiota composition. The hypothesis was that coccidiostat supplementation reduces phytate breakdown through reduction of abundance of phytase-producing bacteria. Since phytase is a frequently used feed supplement and interactions between dietary P, Ca and phytase on measured traits have been observed [4, 24], the coccidiostat effect (Coc) was investigated at different P and Ca (P/Ca) and phytase (Phy) levels. These results could help explaining variations in the results of P metabolism studies. As an additional aspect, the effect of discontinued compared to continuous coccidiostat supplementation on traits measured at the end of the experiment was investigated.

## Results

### Performance traits

The initial bodyweight (BW) of broiler chickens was 42.8 g on d 1, and did not differ between treatments (*P* = 0.805; one-way analysis of variance (ANOVA)). Bodyweight and average daily gain (ADG) at the end of the experiment were affected by the interaction between P/Ca × Phy × Coc (*P* = 0.033 and 0.045, respectively, Table 1). Average daily feed intake (ADFI) was increased by supplements of P/Ca, phytase, and coccidiostat with a significant P/Ca × Phy interaction (*P* < 0.001). Lowest values for each of the three traits were observed in treatment P/Ca-Phy-Coc-, and highest in P/Ca+Phy+Coc+. The P/Ca × Phy interaction was significant for the gain-to-feed ratio (G:F) (*P* < 0.001), with highest values for P/Ca+Phy+ and P/Ca-Phy+, and lowest for P/Ca-Phy-. Each of these traits behaved in a very similar way when only data recorded in phase 2 are considered. In phase 1, both phytase and coccidiostat supplementation increased ADG, ADFI, and G:F.

**Table 1:** Effect of the experimental diets on performance traits of broilers^1^

	Phase 1 (d1-10, *n*=14 pens)			Phase 2 (d10-24/25)			Phase 1+2 (d1-24/25)			d24/25
	ADG	ADFI	G:F	ADG	ADFI	G:F	ADG	ADFI	G:F	BW
	g/d	g/d	g/g	g/d	g/d	g/g	g/d	g/d	g/g	g
P/Ca-Phy-Coc-	-*	-	-	35^e^	50	0.71	28^f^	37	0.76	703^d^
P/Ca-Phy-Coc+	-	-	-	40^d^	56	0.72	32^e^	41	0.77	785^d^
P/Ca-Phy+Coc-	-	-	-	56^bc^	72	0.78	42^bc^	51	0.82	1018^bc^
P/Ca-Phy+Coc+	-	-	-	58^b^	74	0.78	43^b^	53	0.82	1049^b^
P/Ca+Phy-Coc-	17	17	0.99	53^c^	68	0.78	39^d^	48	0.81	965^c^
P/Ca+Phy-Coc+	18	18	1.01	54^c^	70	0.77	40^cd^	50	0.80	979^c^
P/Ca+Phy+Coc-	18	18	1.02	58^b^	73	0.79	43^b^	52	0.82	1046^b^
P/Ca+Phy+Coc+	19	19	1.03	64^a^	80	0.80	47^a^	57	0.83	1143^a^
P/Ca-Phy-Coc±^2^	-	-	-	37	52	0.71	30	39	0.77	752
pooled SEM	0.3	0.3	0.005	1.3	1.7	0.006	1.0	1.2	0.005	26.1
	*P-*values									
*P/Ca*	-*	-	-	<0.001	<0.001	<0.001	<0.001	<0.001	<0.001	<0.001
*Phytase*	<0.001	<0.001	<0.001	<0.001	<0.001	<0.001	<0.001	<0.001	<0.001	<0.001
*Coccidostat*	0.001	0.006	<0.001	0.001	<0.001	0.662	0.001	<0.001	0.962	0.001
*P/Ca×Phy*	-	-	-	<0.001	<0.001	<0.001	<0.001	<0.001	<0.001	<0.001
*P/Ca×Coc*	-	-	-	0.932	0.748	0.237	0.910	0.692	0.184	0.962
*Phy×Coc*	0.849	0.749	0.441	0.536	0.725	0.803	0.588	0.738	0.825	0.600
*P/Ca×Phy×Coc*	-	-	-	0.030	0.083	0.069	0.045	0.099	0.108	0.033

^1^*ADG* average daily gain, *ADFI* average daily feed intake, *G:F* feed consumption, *BW* bodyweight; *n* = 7 pens unless otherwise stated

^2^Additional treatment, was not part of the three-factorial analysis

*Only P/Ca+ treatments in phase 1

^a–f^Means within a column not showing a common superscript differ (*P* ≤ 0.05)

### P, Ca and crude protein digestibility, InsP_6_ disappearance, and foot ash

Precaecal P digestibility was increased by phytase supplementation, at P/Ca- to a greater extent (28.7 percentage points) than at P/Ca+ (17.8 percentage points), which resulted in a P/Ca × Phy interaction (*P* < 0.001; Table 2). Precaecal Ca digestibility was affected by the P/Ca × Coc interaction (*P* = 0.024), but P/Ca- showed clearly higher values for both levels of coccidiostat supplementation. Precaecal crude protein (CP) digestibility was significantly affected by an interaction between P/Ca × Phy × Coc (*P* = 0.050). The lowest CP digestibility was observed for treatment P/Ca-Phy+Coc- (76.2 %), and the highest for P/Ca+‍Phy+Coc+ (82.4 %). The combined supplementation of phytase and coccidiostat had an increasing effect on precaecal CP digestibility at both P/Ca levels.

**Table 2:** Effect of the experimental diets on precaecal nutrient digestibility, InsP_6_ disappearance^1^, and foot ash^2^

	P digestibility	Ca digestibility	CP digestibility	Crop InsP_6_ disappearance	Ileum InsP_6_ disappearance	Foot ash	Foot ash
	%	%	%	%	%	mg	% of DM
P/Ca-Phy-Coc-	47.1	64.0	77.6^de^	-1.7	47.7	568^e^	9.4^f^
P/Ca-Phy-Coc+	48.0	61.1	77.5^de^	4.5	48.6	639^d^	9.9^e^
P/Ca-Phy+Coc-	76.1	62.8	76.2^e^	71.4	88.0	1079^c^	13.3^d^
P/Ca-Phy+Coc+	76.4	60.1	81.6^abc^	80.5	86.6	1074^c^	13.1^d^
P/Ca+Phy-Coc-	45.7	39.9	79.7^bcd^	-0.7	11.6	1146^b^	14.4^b^
P/Ca+Phy-Coc+	46.5	44.6	82.1^ab^	2.8	12.6	1077^c^	13.9^c^
P/Ca+Phy+Coc-	63.0	39.3	79.5^cd^	63.9	77.3	1277^a^	14.9^a^
P/Ca+Phy+Coc+	64.8	39.5	82.4^a^	70.9	76.2	1281^a^	14.8^a^
P/Ca-Phy-Coc±^3^	47.3	63.1	76.5	-0.1	50.3	606	9.5
pooled SEM	1.92	1.54	1.28	5.51	2.77	22.2	0.12
	*P-*values						
*P/Ca*	<0.001	<0.001	<0.001	0.286	<0.001	<0.001	<0.001
*Phytase*	<0.001	0.079	0.277	<0.001	<0.001	<0.001	<0.001
*Coccidostat*	0.489	0.854	<0.001	0.126	0.927	0.977	0.257
*P/Ca×Phy*	<0.001	0.437	0.282	0.327	<0.001	<0.001	<0.001
*P/Ca×Coc*	0.793	0.024	0.968	0.770	0.970	0.013	0.024
*Phy×Coc*	0.937	0.354	0.022	0.696	0.565	0.940	0.525
*P/Ca×Phy×Coc*	0.757	0.295	0.050	0.964	0.982	0.005	0.002

^1^*n*=7 pens

^2^*n*=70 birds

^3^Additional treatment, was not part of the three-factorial analysis

^a–e^Means within a column not showing a common superscript differ (*P* ≤ 0.05)

In the crop, InsP_6_ disappearance was increased by phytase supplementation by 70.5 percentage points (*P* < 0.001). For the InsP_6_ disappearance up to the terminal ileum, the P/Ca x Phy interaction was significant (*P* < 0.001) with greater disappearance found at the low P/Ca level. Coccidiostat supplementation had no effect. The highest value was observed for P/Ca-Phy+ (87.3 %), lowest for P/Ca+Phy- (12.1 %). For foot ash, either expressed as total amount or percentage, the three-way interaction was significant (*P* = 0.005 and 0.002, respectively). Both P/Ca and phytase supplementation increased food ash. Coccidiostat increased foot ash at P/Ca-, but decreased it at the higher P/Ca level in the absence of phytase.

### Blood metabolites

In blood serum, inorganic phosphate (P_i_) and Ca concentrations were influenced by the interaction between P/Ca × Phy, but not by coccidiostat (Table 3). The P_i_ concentration was increased by both P/Ca and phytase supplementation. In contrast, Ca was highest in P/Ca-Phy- and decreased upon P/Ca and phytase supplementation. Serum alkaline phosphatase (ALP) activity was decreased by P/Ca supplementation (*P* < 0.001). The significant interaction between Phy × Coc (*P* = 0.044) was caused by a decreased ALP level with supplementation of coccidiostat, phytase, or both. *Myo*-inositol (MI) in blood plasma was not affected by coccidiostat supplementation, but decreased by P/Ca (by 0.06 mmol/l; *P* < 0.001) and increased by phytase supplementation (by 0.15 mmol/l; *P* < 0.001).

**Table 3:** Effect of the experimental diets on P_i_, Ca, ALP and *myo*-inositol in the blood^1^

	P_i_	Ca	ALP	*Myo*-inositol
	mmol/l	mmol/l	U/l	mmol/l
P/Ca-Phy-Coc-	1.2	3.1	9239	0.33
P/Ca-Phy-Coc+	1.4	3.3	6525	0.31
P/Ca-Phy+Coc-	2.2	2.6	7368	0.48
P/Ca-Phy+Coc+	2.5	2.6	6281	0.40
P/Ca+Phy-Coc-	2.6	2.6	6753	0.23
P/Ca+Phy-Coc+	2.5	2.7	4292	0.22
P/Ca+Phy+Coc-	2.7	2.5	4745	0.39
P/Ca+Phy+Coc+	2.9	2.6	4407	0.43
P/Ca-Phy-Coc±^2^	1.2	2.9	8457	0.30
pooled SEM	0.09	0.12	657.7	0.023
	*P-*values			
*P/Ca*	<0.001	<0.001	<0.001	<0.001
*Phytase*	<0.001	<0.001	0.031	<0.001
*Coccidostat*	0.056	0.231	0.001	0.296
*P/Ca×Phy*	<0.001	0.001	0.904	0.071
*P/Ca×Coc*	0.173	0.954	0.586	0.054
*Phy×Coc*	0.199	0.412	0.044	0.851
*P/Ca×Phy×Coc*	0.515	0.397	0.787	0.082

^1^P_i_: inorganic phosphate, Ca: calcium and ALP: alkaline phosphatase in blood serum; *myo*-inositol in blood plasma; *n*=14 birds

^2^Additional treatment, was not part of the three-factorial analysis

### Inositol phosphates and pH in crop and ileal digesta

The pH in crop digesta was not affected by the experimental diets (Additional file 1: Table S1). The concentration of Ins(1,2,3,4,5)P_5_ in crop digesta was decreased by phytase supplementation, at P/Ca- to a higher extend than at P/Ca+, and increased by coccidiostat supplementation when phytase was not added (*P*‍ =‍ 0.020). Phytase supplementation decreased InsP_6_ and Ins(1,2,4,5,6)P_5_ concentrations, but increased concentrations of InsP_3x_ and Ins(1,2,5,6)P_4_ in crop digesta. Ins(1,2,4,5,6)P_5_, Ins(1,2,3,4,5)P_5_ and Ins(1,2,5,6)P_4_ were the only InsP_4-5_ analysed in crop digesta, whereas further isomers of these InsPs occurred in ileal digesta (Additional file 1: Table S2). In ileal digesta, the P/Ca × Phy interaction was significant for InsP_6_ and Ins(1,2,4,5,6)P_5_ (*P* < 0.001). Both were increased by P/Ca and decreased by phytase supplementation. The remaining isomers, except Ins(1,5,6)P_3_, were increased by P/Ca supplementation. Phytase supplementation decreased Ins(1,2,3,4,6)P_5_ and increased Ins(1,2,5,6)P_4_ and InsP_3x_ concentrations. The coccidiostat alone had no effect on InsPs degradation but increased concentration of InsP_3x_ (*P* = 0.049) in combination with phytase. Ileal pH was increased by 0.4 by both P/Ca and coccidiostat supplementation. The MI concentration in ileal digesta was significantly affected by P/Ca x Phy interaction (*P*‍‍ = 0.013). P/Ca supplementation reduced ileal MI concentration (by 1.3 – 2.0 g/kg DM), while phytase increased it (by 1.2 – 1.9 g/kg DM). There was a trend towards coccidiostat addition increasing ileal MI (*P* = 0.090).

### Microbial communities in the crop and ileum

**Fig. 1:**
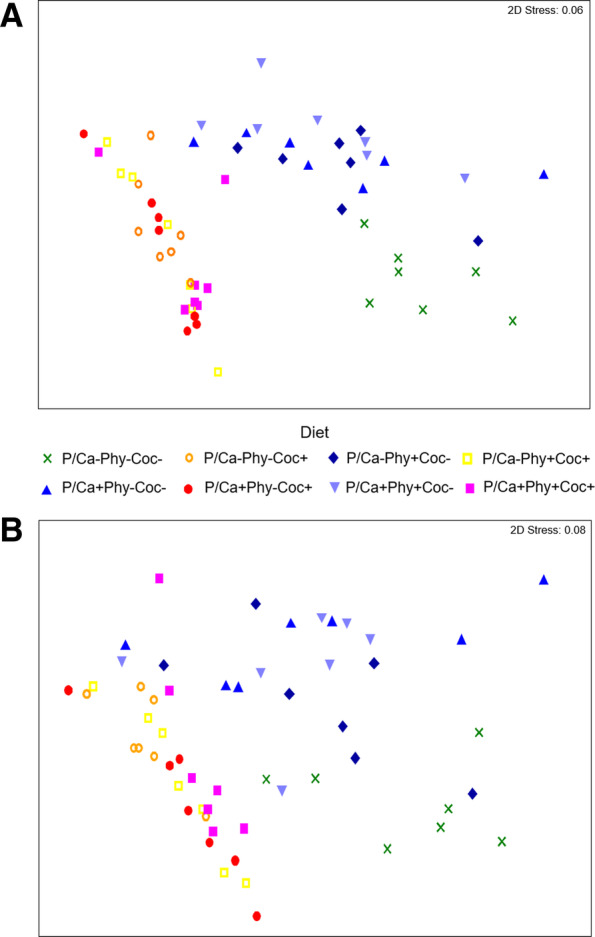
Non-metric multi-dimensional scaling plot. The global bacterial community structure of dietary treatments in the digesta samples of crop (**a**) and ileum (**b**). The symbols represent one pooled sample from each pen comprising all Operational Taxonomic Units

The microbial composition was significantly different between crop and ileum (*P* = 0.001) and between the eight dietary treatments in both crop and ileum (*P* < 0.001) (Additional file 1: Table S3A). Specifically, for both ileum and crop, the treatments supplemented with coccidiostat were significantly different from the non-supplemented (*P* = 0.001, Fig. 1a and b). The interaction section × treatment was not significant.

A total of 592 operational taxonomic units (OTUs) were shared between crop and ileum, 62 OTUs appeared only in the crop and 27 only in the ileum (Additional file 1: Figure S1A). The ten most abundant OTUs were detected in both sections and taken together they accounted for a relative abundance ranging from 95.7 % (P/Ca+Phy-Coc- ileum) to 99.1 % (P/Ca-Phy+Coc- crop). Within these OTUs, the phylum *Firmicutes* and the genus *Lactobacillus* were identified as the most abundant.

**Fig. 2:**
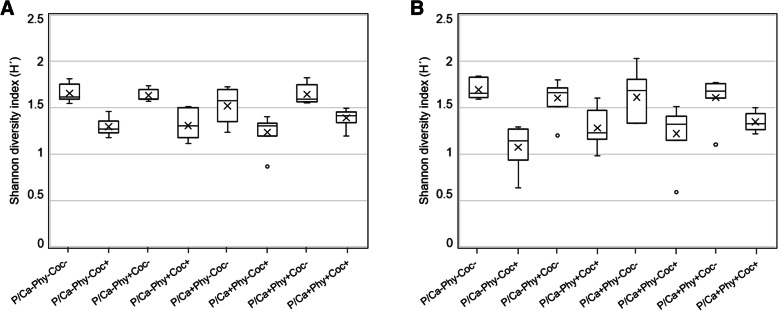
Diversity measured through the Shannon diversity index. Box plots correspond to the crop (**a**) and ileum (**b**) digesta samples

The P/Ca × Coc interaction was significant (*P* = 0.008) for the crop and the P/Ca × Phy × Coc interaction for the ileum (*P* = 0.044, Additional file 1: Table S3A). In both sections, three main groups were observed at a similarity percentage of 76 % in the crop and 70 – 76 % in the ileum (Fig. 1a and b, Additional file 1: Figure S2A and B). The first group comprised mainly samples from treatment P/Ca-Phy-Coc-, the second group all treatments supplemented with coccidiostat, and the third group consisted of treatments without coccidiostat supplementation, but with the high level of P/Ca or phytase or both. Coccidiostat supplementation resulted in a lower diversity (Shannon-Weaver index (H´)) in the microbial composition in crop and ileal digesta compared to treatments without coccidiostat (Fig. 2).

The number of OTUs differed between the treatments. In the crop, 121 (18.5 %) OTUs were detected as part of the core microbiota shared between all treatments (Additional file 1: Figure S1B). Treatment P/Ca-Phy-Coc+ had the lowest number of OTUs (334), whereas treatment P/Ca-Phy-Coc- had the largest (416). In the ileum, 86 OTUs (13 %) were commonly present across all treatments (Additional file 1: Figure S1C). The lowest amount of detected OTUs was found in treatment P/Ca-Phy+Coc+ (264), and the highest was detected in treatment P/Ca+Phy-Coc- (333).

**Fig. 3:**
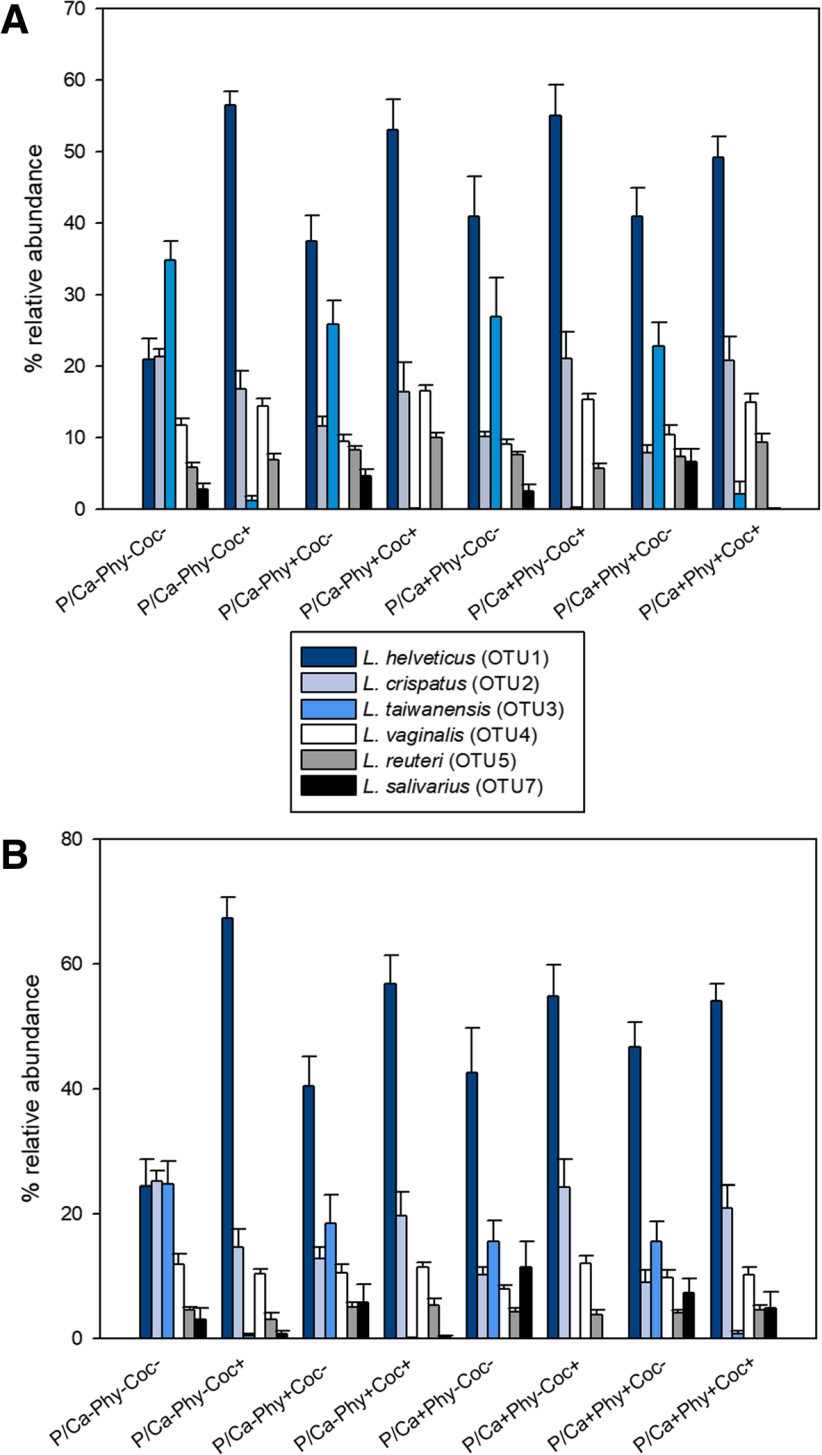
Relative abundance of most abundant Operational Taxonomic Units (OTUs). The assigned OTUs correspond to *Lactobacillus* genus, found in the crop (**a**) and ileum (**b**) digesta samples. Error bars indicate standard deviation of the mean

*Lactobacillus helveticus* (OTU1) was the most dominant OTU in both sections. Birds were colonized in higher abundance by this OTU when the coccidiostat was supplemented (Fig. 3). The highest abundance (56.5 % in the crop and 67.4 % in the ileum) occurred in treatment P/Ca-Phy-Coc+. Among the treatments without coccidiostat supplementation, the highest abundance of OTU1 was detected in treatment P/Ca+Phy+Coc- with 41 % in the crop and 46.7 % in the ileum. *Lactobacillus crispatus* (OTU2) was the second most abundant OTU in treatments with coccidiostat supplementation, with the highest abundance in treatment P/Ca+Phy-Coc+ (21 % in crop and 24.3 % in ileum). In treatments without coccidiostat supplementation, *Lactobacillus taiwanensis* (OTU3) was the second most abundant OTU in the crop and the ileum (with exception of P/Ca-Phy-Coc-). For this OTU, the highest abundance was observed in treatment P/Ca-Phy-Coc- with 35 % in the crop and 25 % in the ileum. The abundance of *Lactobacillus vaginalis* (OTU4) in the crop was higher in treatments with coccidiostat supplementation compared to those without. The abundance of *Lactobacillus reuteri* (OTU5) seems not to follow a specific pattern, while *Lactobacillus salivarius* (OTU7) was decreased in both ileum and crop upon coccidiostat supplementation.

### Effects of discontinued coccidiostat supplementation

When birds were provided with a diet containing the coccidiostat only in phase 1 (treatment P/Ca-Phy-Coc±), they did not significantly differ from the treatment P/Ca-Phy-Coc- in any of the analyzed traits and in microbiota composition at the end of the experiment (Additional file 1: Figure S3, Table 3B and Additional file 1: Table S4, S5 and S6). However, measured P_i_ and Ca in blood serum, foot ash, MI in the terminal ileum, crop InsP_6_ disappearance, and Ins(1,2,3,4,5)P_5_ concentration were significantly lower in treatment P/Ca-Phy-Coc± than in P/Ca-Phy-Coc+. Microbial communities significantly differed between treatments P/Ca-Phy-Coc- or P/Ca-Phy-Coc± and P/Ca-Phy-Coc+.

The microbial communities had a similar distribution in the non-metric multidimensional scaling plots (nMDS) for the treatments P/Ca-Phy-Coc- and P/Ca-Phy-Coc±, but not for treatment P/Ca-Phy-Coc+ (Additional file 1: Figure S3). Differences in the abundance were mainly observed for OTU1 and OTU3, which were particularly dominant in crop and ileum. The abundance of *L. helveticus* (OTU1) was higher in treatment P/Ca-Phy-Coc+ (57 % in crop and 67 % in ileum) compared to P/Ca-Phy-Coc- (21 % in crop and 25 % in ileum) and P/Ca-Phy-Coc± (31 % in the crop and 35 % in ileum; Additional file 1: Figure S4). *L. taiwanensis* (OTU3) was significantly more abundant in treatments P/Ca-Phy-Coc± (31 % in crop and 20.4 % in ileum) and P/Ca-Phy-Coc- (35 % in the crop and 25 % in the ileum) than in treatment P/Ca-Phy-Coc+ (< 1 % in both crop and ileum).

## Discussion

This study investigated for the first time interactive effects of coccidiostat, phytase, and P/Ca supplementation on phytate degradation and related traits. Dietary phytase supplementation increased InsP_6_ degradation while P/Ca supplementation reduced it, which has been observed and discussed before [3, 4, 25]. Therefore, the subsequent discussion will focus on effects of the coccidiostat supplementation and the interactions with P/Ca and phytase.

### Phytate degradation

The hypothesis that coccidiostat supplementation reduced the abundance of phytase producing microorganisms and thus phytate breakdown in the digestive tract of broilers has to be rejected. Substantial differences in microbiota composition between treatments were found, especially between the diets supplemented with coccidiostat or not. It is likely that the change in the composition was caused by the ionophore agent of the supplemented coccidiostat product. In contrast to nicarbazin, ionophore coccidiostats like narasin have antibacterial effects [11–13, 26]. However, coccidiostat supplementation did not significantly affect InsP_6_ disappearance, precaecal P digestibility, P_i_ in blood serum, and concentration of most of the InsP isomers in crop and ileum digesta. The coccidiostat supplementation tended to increase MI concentration in the ileum, suggesting that the coccidiostat may have had an effect on intestinal phosphatases. Nevertheless, precaecal P digestibility was unaffected, indicating that any effect on phosphatases was not of much relevance.

The existence of interactions between the chicken intestinal microbiota and the diet is well described [27–29]. Nonetheless, there are only few studies using next-generation sequencing to investigate the effect of a coccidiostat on the caecal microbiota in chickens. In one study, monensin decreased the abundance of OTUs from the genus *Lactobacillus*, *Enterococcus,* and *Roseburia*, while it increased the abundance of *Coprococcus* and *Anaerofilum* [12]. Also in the present study, significant changes in some *Lactobacillus* species, which was the most abundant genus, were detected. Phytase activity has been reported to exist in different strains of *Lactobacillus, e.g., in L. plantarum, L. fermentum, L. sanfrancisensis, L. reuteri* and *L. salivarius* [14, 30–33]. In the NCBI database, a coding region comprising the phytase gene (accession Nr: KQ961566) in a strain of *L. crispatus* isolated from humans was found. In the present study, *L. helveticus* was more abundant in the crop and ileum when diets contained the coccidiostat supplementation while *L. taiwanensis* and *L. salivarius* were decreased in abundance. *L. crispatus* only increased with coccidiostat supplementation at P/Ca+ and *L. vaginalis* only in the crop. The abundance of *L. reuteri* was not affected by coccidiostat supplementation. Of note, although the abundance of species with potential phytase activity was changed, this was not reflected by changes in phytate degradation. Therefore, it is possible that either the major phytase producing microorganisms were not affected by coccidiostat supplementation, or phytase producing microorganisms have generally a very restricted influence on phytate degradation, or compensatory mechanisms were involved. Potential compensatory mechanisms could be an increasing abundance of other phytase producing species to compensate for those that were decreased, an intensification of mucosa-derived phytase activity, or a higher phytate degradation activity of those bacterial species remaining to exist in the presence of coccidiostats.

The relevance of mucosa-derived phytase for phytate degradation in the gut lumen is supposed to be low because of their localization in the brush-border membrane [5]. However, in a study with gnotobiotic broiler chickens, substantial amounts of InsP_6_ disappeared until the end of the ileum when a low P/Ca diet without phytase supplementation was fed [6]. This points towards a high contribution of mucosa derived phytase to precaecal InsP_6_ disappearance. In the present study, the pattern of InsP isomers was only marginally influenced by coccidiostat supplementation, suggesting that the steps of phytate degradation and involved phytase producers did not change. Perhaps, mucosa-derived phytase overall contributes more to InsP_6_ degradation than microbiota-derived phytase as often assumed.

Another explanation for the lack of effects on InsP_6_ disappearance could be a higher efficiency of remaining bacterial species. Species diversity was lower in the crop and ileum when the coccidiostat was supplemented. In a study using kakapo birds’ faeces, microbial diversity and abundance of cellulolytic microorganisms were low, but degradation of cellulose substrates was high [34]. These authors concluded that taxonomic diversity alone does not accurately reflect the ‘true’ functional diversity within an ecosystem. In cows, a high feed efficiency was found to be related to a lower richness of the rumen microbiome [35]. In obese humans, low microbial diversity in faecal samples was coupled with high energy use from the food [36]. Therefore, the decrease of the microbiota diversity in the present study could have implied positive effects for the host and lead to higher phytase activity of the remaining microorganisms. Such mechanism would help to explain that the reduction in microbial diversity with coccidiostat administration was not coupled with an affect phytate degradation.

Although precaecal InsP_6_ disappearance and P digestibility were not influenced by coccidiostat supplementation, ALP activity in blood serum was decreased by P/Ca, phytase, and coccidiostat supplementation. Increased ALP activity in the serum is associated with skeletal disorders or liver dysfunction and may be related to Ca or P deficiency or an undesirable Ca:P ratio in the diet [37, 38]. A down-regulation of this enzyme with increased availability of P has been reported [39]. This relationship was confirmed by the present study, where negative correlations between ALP activity and P_i_ in blood serum (r = -0.489, *P* < 0.001), and between ALP and foot ash amount (r = -0.611, *P* < 0.001) existed. It remains unclear though that the coccidiostat supplementation increased bone ash at P/Ca- but reduced it at P/Ca+. Because this coccidiostat effect disappeared in the presence of phytase, it is likely that animals might be more sensitive to P/Ca imbalances in the presence of the coccidiostats. The higher ileal pH in the presence of the coccidiostat might have reduced mineral solubility, possibly by calcium phosphate precipitation.

### Crude protein digestibility

In the present study, CP digestibility values were increased by coccidiostat supplementation when more P was available, either due to phytase or mineral P supplementation. A similar effect was observed by McCormick et al. [21]. They found a significantly increased precaecal N digestibility in P deficient diets when phytase and the antibacterial products tylosin or virginiamycin were supplemented, but no single effect of phytase or the antimicrobials.

Effects of phytase supplementation on precaecal CP and amino acid (AA) digestibility exist, but effects are not consistent in literature. Explanations for phytase effect on AA digestibility include the release of protein from protein-phytate complexes and the reduction of negative impact of phytate on digestive enzymes [40]. In the present study, AAs were not analyzed, but the precaecal CP digestibility was not increased by the phytase supplementation alone. Increased CP digestibility as a result of phytase being more effective in reducing phytate-protein-complexes in the presence of coccidiostat seems unlikely. If this was the case, InsP_6_ disappearance would increase when both products were used together. Of note, the coccidiostat supplementation significantly increased pH in ileal digesta from 6.3 to 6.7, which was highly correlated with precaecal CP digestibility (r = 0.816; *P* < 0.001). This increment in pH probably did not support phytate hydrolysis because the supplemented phytase acts most efficient within an optimum pH of 3.5-5.0 [41] and the pH in the lower small intestine is less relevant for phytase. Also, increased ileal pH will promote phytate precipitation which is contrasting to the observations on InsP degradation. Therefore, it is more likely that the observed coccidiostat effect was caused by a decrease in microbial protein production, leading to an increased precaecal CP digestibility. In treatments that included the coccidiostat supplementation, negative correlations between the CP digestibility and the presence of some OTUs (2, 3, 4, 5, and 7, assigned to *L. crispatus, L. taiwanensis, L. vaginalis, L. reuteri,* and *L. salivarius*) were observed. In the low P/Ca treatment without phytase, coccidiostat supplementation did not affect CP digestibility. Possibly a lack of P in the P/Ca reduced diets without phytase supplementation made it impossible for the birds to absorb more AA due to the importance of phosphate for membrane function and transporters like the Na/K-ATPase pump, which are necessary for AA absorption [42].

Another explanation for the increased CP digestibility could be a reduction of endogenous losses by coccidiostat and phytase supplementation. Coccidiostat supplementation may decrease endogenous losses for instance by extending the digesta retention time [43] or affecting the activity of digestive enzymes [44, 45]. Phytate is known to increase mucin production and endogenous AA losses [46, 47]. This effect was shown to be reduced by phytase supplementation [46, 48]. The combination of coccidiostat and phytase supplementation and the high availability of P could have led to the highest observed CP digestibility in treatment P/Ca+Phy+Coc+ resulting in the significant interaction between those supplements. Because our attempts to explain the effects on CP digestibility were speculative, more experiments should be done using different coccidiostats and phytase supplements and including AA analysis of the digesta. Further, it should be determined if the effect of coccidiostats on precaecal CP digestibility is correlated with microbial protein production.

### Effect of discontinued coccidiostat supplementation

The additional treatment P/Ca-Phy-Coc± was implemented to study the effect of a coccidiostat supplement only during the starter phase on traits measured at the end of the experiment. This treatment contained the coccidiostat in phase 1, but not in phase 2 and was therefore compared to treatments P/Ca-Phy-Coc- and P/Ca-Phy-Coc+. Microbial abundance and diversity, and all other traits did not differ between the treatments P/Ca-Phy-Coc± and P/Ca-Phy-Coc- at the end of the experiment. This is remarkable considering that the early phase post-hatch is important for establishment of the gut microbial community in broiler chickens. At the time of hatching, the gastrointestinal environment is nearly sterile and with ageing, the microbial population increases and becomes more complex [44, 49]. In the present study, results indicated that the coccidiostat supplementation in phase 1 did not influence the microbial composition, or that the microbiota has adapted during phase 2 after the removal of the coccidiostat. Coccidiostat supplementation increased performance traits during phase 1. This may be seen to contradict the view of the coccidiostat not having an effect in early stage post hatch.

Significant differences in microbiota composition existed between treatments P/Ca-Phy-Coc± and P/Ca-Phy-Coc+, but not between P/Ca-Phy-Coc± and P/Ca-Phy-Coc-. For some other traits, in particular ADG, ADFI, and BW during the whole trial, treatment P/Ca-Phy-Coc± was not significantly different from the other two treatments. This indicates again towards an adaptation of the microbiota. A similar observation was made in a pig study, where precaecal AA digestibility was increased when virginiamycin was added to the diets, potentially induced by changes in the microbiota [50]. After removal of virginiamycin from the diet, this effect did no longer exist.

Based on our results, it is not expected that a mixture of Narasin and Nicarbazin in the early phase of the experiment has an influence on microbiota and P digestibility at the end of the experiment.

## Conclusions

We conclude that a mixture of the coccidiostats Narasin and Nicarbazin had no discernible effect on endogenous phytase activity in the digestive tract anterior to the caeca. Coccidiostat supplementation changed the microbial distribution and diversity in the digestive tract of broilers, but did not affect phytate breakdown. Coccidiostat supplementation confined to the early phase of the experiment had no influence on microbiota and P digestibility at the end of the experiment. Crude protein digestibility was increased by coccidiostat supplementation when more P was available. Effects of supplemented phytase were not influenced by coccidiostat supplementation. These results should be verified using other coccidiostats. Further work is needed to investigate if the effect of coccidiostats on precaecal CP digestibility is correlated with microbial protein production. The influence of microbiota-derived phytase on phytate degradation processes needs to be elucidated.

## Methods

### Birds and housing

The trial was performed in accordance with the German Animal Welfare Legislation, approved by the Regierungspräsidium Tübingen, Germany (project no. HOH 46/17 TE) and conducted at the Agricultural Experiment Station of the University of Hohenheim. A total of 630 male Ross 308 broiler hatchlings were supplied by a commercial hatchery (*Brüterei Süd GmbH & Co. KG*, Regenstauf, Germany) and assigned to one of 9 treatments with 7 pens each in a completely randomized block design. Each floor pen (115 × 230 cm ground area, 260 cm high) was stocked with 10 hatchlings. Feed and tap water were provided for *ad libitum* consumption during the whole trial. Birds were kept on deep litter bedding until d 14. From then they were kept on perforated floors until the end to avoid an intake of litter or excreta that contain the indigestible marker. The lighting program was 24 h light : 0 h darkness until d 3, and from then 18 h light : 6 h darkness. The temperature was set at 34 °C on the day of placement and continuously decreased to achieve a temperature of 26 °C on the last day. The well-being of the animals was checked at least twice daily.

### Diets and treatments

Birds were fed corn-soybean meal-based diets in 2 phases (day 1-10 and 10-25). Diets were based on the recommendations of the Gesellschaft für Ernährungs-physiologie (GFE, 1999) [51] with the exception of P and Ca in phase 2 (Table 4). The experiment was designed as a 2 × 2 × 2 + 1-factorial arrangement of treatments. It included diets without (P/Ca−, 4.2 g P and 6.5 g Ca/kg DM in phase 2) or with monocalcium phosphate and adjusted limestone supplementation (P/Ca+, 7.0 g P and 10.4 g Ca/kg DM in phase 2), without (Coc-) or with coccidiostat supplementation (Coc+, 50 mg/kg of Narasin and Nicarbazin each; Maxiban®, Elanco, Greenfield, USA), and without (Phy−) or with a modified, *E. coli*-derived 6-phytase (Phy+, 1,500 FTU/kg feed; Quantum Blue^TM^, AB Vista, Marlborough, UK). In phase 1, P/Ca concentrations of all diets were according to the recommendations of GfE (1999) [51]. Coccidiostat and phytase were supplemented continuously to the respective diets in phase 1 and 2. An additional treatment (P/Ca-Phy-Coc±) was implemented which contained the coccidiostat in phase 1, but not in phase 2. This treatment intended to study the effect of a coccidiostat fed only during the starter phase on results at the end of the experiment.

**Table 4:** Ingredient composition of the diets and calculated concentrations

Ingredient, g/kg	Phase 1 (d 1-10)	Phase 2 (d 10-24/25)	
	P/Ca+^1^	P/Ca-^1^	P/Ca+^1^
Corn	550	575	575
Soybean Meal	370	350	350
Soy Crude Oil	30	30	30
Monocalcium Phosphate	17	-	11
Limestone	16	11	16
Sand	-	16	-
Vitamin Premix^2^	2	2	2
Mineral Premix^3^	0.5	0.5	0.5
DL-Methionine	2	3.5	3.5
Sodium Bicarbonate	3	3	3
Sodium Chloride	1.5	1	1
Choline Chloride	2	2	2
TiO_2_	5	5	5
Calculated composition, g/kg DM			
Crude Protein	237	230	230
Total Phosphorus (tP)	8.5	4.2	7.0
Calcium	11.5	6.5	10.4
Ca:tP	1.3	1.5	1.5

^1^Includes treatments Phy-Coc-, Phy-Coc+, Phy+Coc-, and Phy+Coc+, where Phy- = 0 and Phy+ = 1500 FTU phytase/kg, Coc- = 0 and Coc+ = 50 mg/kg of Narasin and Nicarbazin each in exchange for sand

^2^Vitamin premix (Miavit GmbH, Essen, Germany), provided per kg of complete diet: 10 000 IU vitamin A, 3000 IU vitamin D3, 30 mg vitamin E, 2.4 mg vitamin K3, 100 mcg biotin, 1 mg folic acid, 3 mg vitamin B1, 6 mg vitamin B2, 6 mg vitamin B6, 30 mcg vitamin B12, 50 mg nicotinamide, 14 mg calcium-D-pantothenat

^3^Trace element premix (Gelamin Gesellschaft für Tierernährung mbH, Memmingen, Germany), provided per kg of complete diet: 25 mg calcium from carbonate, 80 mg manganese from manganese-(II)-oxide, 60 mg zinc from zinc-oxide, 25 mg iron from ferrous-(II)-sulphate monohydrate, 7.5 mg copper from cupric-(II)-sulphate pentahydrate, 0.6 mg iodine from calcium iodate, 0.2 mg selenium from sodium selenite

The calculated concentration of ME was 14.0 MJ/kg DM in all diets of phase 2. All phase 2 diets contained 5 g/kg TiO_2_ as an indigestible marker. The experimental diets were produced by first mixing all ingredients of the respective phase with the exception of the variable ingredients. For phase 2, this premix was divided into 2 parts. Both parts were then supplemented with either limestone and monocalcium phosphate or sand, and mixed again. Each of the resulting mixtures and the phase 1 premix was then divided into 4 parts and individually supplemented with a mixture of the exogenous phytase product, the coccidiostat, and sand. Afterwards, the diets were mixed again and pelleted without using steam conditioning at a pelleting temperature below 80 °C, which was checked continuously. Pellet diameter was 2 mm for the phase 1 mixtures, and 3 mm for the phase 2 mixtures. Representative samples of each diet were taken, pulverized by a vibrating cup mill (PULVERISETTE 9, Fritsch GmbH, Idar-Oberstein, Germany) and analyzed. Intended concentrations of P, Ca, phytase and coccidiostat were confirmed by analysis (Table 5).

**Table 5:** Analyzed composition of the experimental diets

	P g/kg DM	Ca g/kg DM	Phytase FTU/kg	Narasin mg/kg	Nicarbazin mg/kg	CP g/kg DM	*Myo*-Inositol g/kg DM	Ins(1,2,3,4,5)P_5_ μmol/g DM	Ins(1,2,4,5,6)P_5_ μmol/g DM	InsP_6_ μmol/g DM
Phase 1										
P/Ca+Phy-Coc-	8.22	11.32	<50	<1	<1	23.5	0.2	0.6	1.1	15.7
P/Ca+Phy-Coc+	8.23	11.39	<50	59	51	24.6	0.2	0.5	1.1	15.8
P/Ca+Phy+Coc-	8.60	11.94	1460	<1	<1	23.8	0.2	0.5	1.0	14.9
P/Ca+Phy+Coc+	8.75	11.53	1570	56	51	24.1	0.2	0.5	1.0	15.0
Phase 2										
P/Ca-Phy-Coc-	4.17	6.53	<50	<1	<1	23.2	0.2	0.5	1.0	15.1
P/Ca-Phy-Coc+	4.18	6.42	<50	59	47	23.2	0.2	0.5	1.1	15.2
P/Ca-Phy+Coc-	4.19	6.48	1520	<1	<1	23.2	0.2	0.5	1.1	15.2
P/Ca-Phy+Coc+	4.20	6.42	1580	60	45	23.3	0.2	0.6	1.2	16.2
P/Ca+Phy-Coc-	7.13	10.86	<50	<1	<1	23.3	0.2	0.5	1.0	15.3
P/Ca+Phy-Coc+	6.84	10.34	<50	56	47	23.1	0.2	0.5	1.0	15.1
P/Ca+Phy+Coc-	6.61	10.11	1600	<1	<1	23.3	0.2	0.5	1.0	15.6
P/Ca+Phy+Coc+	6.57	10.02	1500	60	50	23.6	0.2	0.5	1.0	15.2

### Sampling and measurements

Animals and feeds were weighed on a pen basis before placement, on d 10, and before slaughter to calculate ADFI, ADG and G:F. To standardize intestinal fill, feed was deprived 2 h before slaughtering followed by 1 h *ad libitum* access to feed. On d 24 (36 pens; 4 pens from each treatment) and on d 25 (27 pens; 3 pens from each treatment) animals were stunned with a gas mixture of 35% CO_2_, 35% N_2_, and 30% O_2_. For blood samples, two randomly chosen birds per pen were killed by decapitation. The trunk blood was collected in tubes containing clot activator for serum samples or sodium fluoride and heparin for plasma samples. Blood samples were then centrifuged for 10 min at 2,500 × g to separate the plasma. The remaining 8 anaesthetized birds of each pen were euthanized by CO_2_ asphyxiation. The right foot of each bird was removed and frozen at -20 °C for bone ash analyses. Digesta from the crop and the terminal part of the ileum, defined as the last two thirds of the section between Meckel´s diverticulum and 2 cm prior the ileo-caeco-colonic junction, were collected and pooled on a pen basis. The crop was clamped with an arterial clamp to prevent emptying, then opened and upended. Crop digesta was gently removed with a spatula without scraping the mucosa, mixed, and pH was measured using a spear-tip electrode (InLab® Solids; Mettler-Toledo, Gießen, Germany) and a subsample was collected into tubes for microbiota analysis. From the terminal ileum, approximately 2 cm was taken from each bird for the microbiota analysis. These parts were cut lengthwise, digesta was gently removed with a spatula without scraping the mucosa, pooled and mixed, and pH was measured. Digesta samples for microbiota analysis were immediately stored on ice and later frozen at -80‍ °C until further analysis. The other parts of the terminal ileum were flushed with cold double-distilled water. Digesta samples not determined for microbiota analysis were immediately frozen at −20 °C, freeze-dried, and pulverized. Pulverized samples were stored in airtight containers until further analysis.

### Chemical analyses

Feed samples were analyzed for DM according to the official methods in Germany (Verband Deutscher Landwirtschaftlicher Untersuchungs- und Forschungsanstalten (VDLUFA), method no. 3.1) [52]. Pulverized feed and ileum digesta samples were analyzed for CP using VDLUFA method no. 4.1.1. [52] and for P, Ca, and TiO_2_ using the modified sulfuric and nitric acid wet digestion method of Boguhn et al. [53]. Measurements were done using inductively coupled plasma optical emission spectrometry, described in detail by Zeller et al. [2].

The extraction and measurement of InsP_3–6_ isomers in feed and digesta were carried out using the method of Zeller et al. [2] with slight modifications, described in detail by Sommerfeld et al. [4]. Using this methodology, separation of enantiomers is not possible. Hence, the presentation of results does not distinguish between D- and L-form. Filtrates were analyzed using high-performance ion chromatography and UV detection at 290 nm in an ICS-3000 system (Dionex, Idstein, Germany). Since some specific InsP_3_ isomer standards were not available, theses isomers could not be identified. A clear discrimination between the isomers Ins(1,2,6)P_3_, Ins(1,4,5)P_3_, and Ins(2,4,5)P_3_ was not possible due to co-elution. Thus, the term InsP_3x_ will be used for these isomers with unknown proportions.

For analysis of MI, feed and digesta were analyzed according to Sommerfeld et al. [4]. Measurements were done using an Agilent 5977A gas chromatograph/mass spectrometer (Waldbronn, Germany) with deuterated MI used as an internal standard.

Alkaline phosphatase, Ca and P_i_ in blood serum were analyzed at the IDEXX BioResearch Vet Med Labor GmbH (Ludwigsburg, Germany) with a Beckman Olympus AU480. P_i_ was measured as phosphomolybdate complex and Ca according to the Arsenazo method. For ALP, the method of the International Federation of Clinical Chemistry with 2-amino-2-methyl-1-propanol buffer was used [54].

The right foot of each bird was detached at the *articulatio intertarsalis* including skin, claws, and all adhering tissues after defrosting. Feet were washed with distilled water, first dried for 48 h at 60 °C in a convection oven (VL 115, VWR International GmbH, Darmstadt, Germany), then for 72 h at 103 °C. Subsequently, they were ashed in a muffle furnace (Nabertherm L 40/11/B170, Nabertherm GmbH, Bremen, Germany) for 48 h at 600 °C.

Feed samples were analyzed for phytase activity by AB Vista Laboratories (Ystrad Mynach, UK) using the analytical method of the supplier (pH 4.5, 60 °C) followed by transferring the results to the commonly used FTU by a validated transfer factor.

For analysis of coccidiostat, feed samples were measured with high performance liquid chromatography at the LUFA Speyer (Germany). Narasin was analyzed according to VDLUFA method no. 14.22.1 [52], and Nicarbazin according to method DIN EN 15782 of the European Committee for Standardization [55].

### DNA extraction, illumina amplicon sequencing and data analysis

DNA from crop and ileum digesta samples was extracted with the commercial DNA extraction kit FastDNA^TM^ Spin Kit for Soil (MP Biomedicals LLC, Solon, OH, USA) following the manufacturer’s instructions. DNA was quantified with a NanoDrop^TM^ 2000 spectrophotometer (Thermo Fisher Scientific, Waltham, MA, USA) and stored at -20 °C. Illumina library was prepared according to Kaewtapee et al. [56]. In brief, the V1-2 region of the 16S rRNA gene was amplified in a two-step polymerase-chain reaction (PCR). One microliter of DNA was used as template in the first PCR, where the forward primer contains a six-nucleotide barcode and both primers have sequences complementary to the Illumina adapters. Subsequent, one microliter of the first PCR product was used in a second PCR following the same conditions, where both primers were complemented to the sequences of Illumina multiplexing and Illumina index primers [57]. The amplicons were verified in an agarose gel electrophoresis with a 2 % agarose gel (ROTH Bioenzyme). Purification and normalization were done through the SequalPrep^TM^ Normalization Plate Kit (Thermo Fisher Scientific, Waltham, MA, USA). The amplicons were pooled per Index and a second purification was performed with the MinElute PCR Purification Kit (Qiagen). Samples were sequenced using the 250 bp paired-end sequencing chemistry on an Illumina MiSeq platform.

Raw reads were checked for quality, assembled and aligned using Mothur pipeline tool [58]. The data included 74,662 ± 3,399 sequences per sample. The UCHIME program included in Mothur pipeline was used to identify possible chimeras [59]. Reads were clustered at 97 % identity into 681 OTUs. Only OTU with an average abundance higher than 0.0001 % and a sequence length >250 bp were considered for further analysis. The closest representative was manually identified using seqmatch from the Ribosomal Database Project [60]. Sequences were submitted to the European Nucleotide Archive (study accession number PRJEB28349).

### Calculations and statistical analyses

To calculate ADG and ADFI, birds’ weight gain or feed consumption on a pen basis were divided by days of life of all animals in a pen. Dead animal´s weight and the pen feed consumption up to the day of occurrence were recorded and considered in the calculation of performance traits. The precaecal digestibility of P, Ca, and CP as well as the InsP_6_ disappearance (Y) were calculated with the following equation:

$$ Y\ \left(\%\right)=100-100\ast \left(\ \frac{Ti\  in\ feed}{Ti\  in\ digesta}\ast \frac{y\  in\ digesta}{y\  in\ feed}\ \right) $$

Where y is the concentration of the respective trait and Ti and y are in grams per kilogram DM.

All traits with the exception of the microbiota data were analyzed using a three-way ANOVA with the MIXED procedure of the software package SAS for Windows (version 9.3; SAS Institute Inc., Cary, NC). While treatment effects were taken as fixed, block effects were assumed as random. For each trait, heterogeneity of error variances between treatments, P/Ca-, phytase- or coccidiostat-levels and combinations of these factors were tested and the model with the smallest AIC was used. For all traits analyzed in this experiment, with the exception of blood and bone ash, samples were pooled on a pen basis and therefore the pen was determined as experimental unit. The following model was fitted:

$$ {Y}_{ijkl}=\mu +{\mathrm{s}}_{\mathrm{l}}+{\alpha}_{\mathrm{il}}+{\beta}_{\mathrm{jl}}+{\gamma}_{\mathrm{kl}}+{\left(\alpha \beta \right)}_{\mathrm{ijl}}+{\left(\alpha \gamma \right)}_{\mathrm{ikl}}+{\left(\beta \gamma \right)}_{\mathrm{jkl}}+{\left(\alpha \beta \gamma \right)}_{\mathrm{ijkl}}+{\varepsilon}_{\mathrm{ijkl}} $$

Where: Y_ijk_ = observation of the response variable, μ = general effect, s_l_ = effect of treatment P/Ca-Phy-Coc±, α_il_ = effect of P/Ca supplementation, β_jl_ = effect of phytase supplementation, γ_kl_ = effect of coccidiostat supplementation, all possible interactions among the effects and ε_ijkl_ = residual error. A graphical check of residuals for normal distribution and homogeneity of variance was done. After finding significant effects, simple or marginal means were compared using a multiple t-test. Blood and bone ash were obtained from individual birds, so the bird was considered as the experimental unit. In this case the pen was included as random effect to the model described above. For bone ash, day of drying and ashing were additionally considered as random effects.

The sequencing dataset was statistical analysed using PRIMER software (version 7.0.9, PRIMER-E, Plymouth Marine Laboratory, Plymouth, UK) in a multivariate analysis. The dataset was first standardized by total, then comparisons between samples were done through a sample similarity matrix using the Bray-Curtis coefficient. Intersection matrix to define core microbiota was done based on the R package UpSet [61]. The community similarity structure was depicted through nMDS. Samples were represented as points in low-dimensional 2D space. A hierarchical cluster analysis was done to show the similarity between samples. PERMANOVA, a non-parametric multivariate statistical test, was used to study the significant differences and interactions of P/Ca, phytase and coccidiostat supplementation on the microbial community in crop and ileum and between the sections. The similarity percentage analysis (SIMPER) identified the OTU contribution to the similarity among samples within each treatment. The Shannon-Weaver index of diversity (H’) was used to calculate sample diversity. Differences in the abundance of OTUs between treatments were evaluated using the unpaired Welch’s t-test in Excel that is able to handle unequal variances, unequal sample sizes and non-parametric data [62]. Correlations were estimated with Pearson correlation coefficient (9999 permutations) using PRISM6 (GraphPad Software, CA). For all statistical analyses, significance was declared at *P* ≤ 0.05.

## Additional file

Additional file 1: **Table S1.** Effect of the experimental diets on InsP isomers and pH in crop digesta. **Table S2.** Effect of the experimental diets on InsP isomers, *myo*-inositol and pH in ileum digesta. **Table S3.** Effect of the experimental diets and the sampling section on the microbial composition (PERMANOVA analysis). **Table S4.** Effect of different coccidiostat treatments on performance traits of broilers. **Table S5.** Effect of different coccidiostat treatments on precaecal nutrient digestibility, InsP_6_ disappearance and foot ash. **Table S6.** Effect of different coccidiostat treatments on blood metabolites and pH in digesta. **Figure S1.** Distribution of the Operational Taxonomic Units (OTUs). **Figure S2.** Cluster analysis for crop (A) and ileum (B) digesta samples. **Figure S3.** Non-metric multi-dimensional scaling plot illustrating the global bacterial community structure. **Figure S4.** Relative abundance for more abundant Operational Taxonomic Units (OTUs) in Crop (A) and ileum (B) digesta samples. (DOCX 4972 kb)

## Data Availability

All data generated or analysed during this study are included in this published article and its supplementary information files.

## Abbreviations

ADFI: Average daily feed intake
ADG: Average daily gain
ANOVA: Analysis of variance
BW: Bodyweight
Ca: Calcium
Coc: Coccidiostat
CP: Crude protein
G:F: Gain-to feed ratio
H´: Shannon-weaver index of diversity
InsP_6_: *Myo*-inositol 1,2,3,4,5,6-hexakis(dihydrogen phosphate)
MI: *Myo*-inositol
nMDS: Non-metric multidimensional scaling plot
OTU: Operational taxonomic units
P: Phosphorus
P/Ca: Phosphorus and Calcium
PCR: Polymerase-chain reaction
Phy: Phytase
P_i_: Inorganic phosphate
