# Supplementary material for: Impact of coccidiostat and phytase supplementation on gut microbiota composition and phytate degradation in broiler chickens
Source: Anim Microbiome. 2019 Jun 28;1:5. doi: 10.1186/s42523-019-0006-2 (PMC7803125; doi:10.1186/s42523-019-0006-2)
Supplement: Supplementary file 1 — Table S1. Effect of the experimental diets on InsP isomers and pH in crop digesta. Table S2. Effect of the experimental diets on InsP isomers, myo-inositol and pH in ileum digesta. Table S3. Effect of the experimental diets and the sampling section on the microbial composition (PERMANOVA analysis). Table S4. Effect of different coccidiostat treatments on performance traits of broilers. Table S5. Effect of different coccidiostat treatments on precaecal nutrient digestibility, InsP6 disappearance and foot ash. Table S6. Effect of different coccidiostat treatments on blood metabolites and pH in digesta. Figure S1. Distribution of the Operational Taxonomic Units (OTUs). Figure S2. Cluster analysis for crop (A) and ileum (B) digesta samples. Figure S3. Non-metric multi-dimensional scaling plot illustrating the global bacterial community structure. Figure S4. Relative abundance for more abundant Operational Taxonomic Units (OTUs) in Crop (A) and ileum (B) digesta samples. (DOCX 4972 kb) [file 42523_2019_6_MOESM1_ESM.docx]

Table S1 Effect of the experimental diets on InsP isomers and pH in crop digesta^1^

|  | InsP_3x_^3^ | Ins(1,2,5,6)P_4_ | Ins(1,2,3,4,5)P_5_ | Ins(1,2,4,5,6)P_5_ | InsP_6_ |  | pH crop digesta |
| --- | --- | --- | --- | --- | --- | --- | --- |
| Diet | µmol/g DM | | | | |  |  |
| P/Ca-Phy-Coc- | n.d. | 0.2 | 0.6 | 1.1 | 14.8 |  | 5.1 |
| P/Ca-Phy-Coc+ | n.d. | <LOQ | 0.8 | 1.1 | 14.5 |  | 5.0 |
| P/Ca-Phy+Coc- | 1.4 | 4.5 | 0.3 | 0.2 | 4.3 |  | 5.2 |
| P/Ca-Phy+Coc+ | 1.4 | 4.1 | 0.2 | 0.2 | 3.1 |  | 5.0 |
| P/Ca+Phy-Coc- | n.d. | <LOQ | 0.7 | 1.1 | 15.1 |  | 5.1 |
| P/Ca+Phy-Coc+ | n.d. | <LOQ | 0.7 | 1.1 | 15.0 |  | 5.1 |
| P/Ca+Phy+Coc- | 1.5 | 4.9 | 0.4 | 0.3 | 5.7 |  | 5.0 |
| P/Ca+Phy+Coc+ | 1.4 | 4.6 | 0.3 | 0.2 | 4.4 |  | 4.9 |
| P/Ca-Phy-Coc±^2^ | n.d. | 0.3 | 0.7 | 1.1 | 14.9 |  | 5.2 |
| pooled SEM | 0.18 | 0.24 | 0.04 | 0.04 | 0.85 |  | 0.13 |
|  | *P-*values | | | | | | |
| *P/Ca* | 0.939 | 0.049 | 0.152 | 0.139 | 0.182 |  | 0.728 |
| *Phytase* | - | <0.001 | <0.001 | <0.001 | <0.001 |  | 0.238 |
| *Coccidostat* | 0.789 | 0.181 | 1.000 | 0.341 | 0.276 |  | 0.126 |
| *P/Ca×Phy* | - | - | 0.020 | 0.891 | 0.438 |  | 0.188 |
| *P/Ca×Coc* | 0.789 | 0.805 | 0.230 | 0.891 | 0.947 |  | 0.562 |
| *Phy×Coc* | - | - | 0.020 | 0.495 | 0.432 |  | 0.606 |
| *P/Ca×Phy×Coc* | - | - | 0.230 | 0.891 | 0.911 |  | 0.949 |

^1^ Not shown isomers were not detectable (n.d.) or not quantifiable (<LOQ) in the majority of samples; n=7 pens

^2^Additional treatment, was not part of the three-factorial analysis

^3^ At least one of the following isomers: Ins(1,2,6)P_3_, Ins(1,4,5)P_3_, Ins(2,4,5)P_3_

Table S2 Effect of the experimental diets on InsP isomers, *myo*-inositol and pH in ileum digesta^1^

|  | InsP_3x_^3^ | Ins(1,5,6)P_3_ | Ins(1,2,3,4)P_4_ | Ins(1,2,5,6)P_4_ | Ins(1,2,3,4,5)P_5_ | Ins(1,2,3,4,6)P_5_ | Ins(1,2,4,5,6)P_5_ | InsP_6_ | |  | *My*o-inositol | pH ileum digesta |
| --- | --- | --- | --- | --- | --- | --- | --- | --- | --- | --- | --- | --- |
| Diet | µmol/g DM | | | | | | | |  | | g/kg DM |  |
| P/Ca-Phy-Coc- | n.d. | 0.2 | 0.3 | n.d. | 1.2 | 0.6 | 0.4 | 24.6 | |  | 1.4 | 6.2 |
| P/Ca-Phy-Coc+ | n.d. | n.d. | 0.2 | n.d. | 1.1 | 0.5 | 0.4 | 25.4 | |  | 1.6 | 6.6 |
| P/Ca-Phy+Coc- | n.d. | <LOQ | n.d. | 0.6 | 0.8 | n.d. | 0.3 | 6.1 | |  | 3.2 | 5.9 |
| P/Ca-Phy+Coc+ | 0.2 | <LOQ | n.d. | 1.1 | 0.8 | n.d. | 0.3 | 7.5 | |  | 3.6 | 6.7 |
| P/Ca+Phy-Coc- | 0.2 | <LOQ | 0.4 | 0.5 | 2.3 | 1.0 | 2.6 | 47.2 | |  | 0.2 | 6.4 |
| P/Ca+Phy-Coc+ | n.d. | 0.2 | 0.3 | 0.5 | 2.3 | 1.0 | 2.5 | 48.4 | |  | 0.2 | 7.0 |
| P/Ca+Phy+Coc- | 2.9 | <LOQ | 0.4 | 5.8 | 2.7 | n.d. | 1.0 | 12.7 | |  | 1.2 | 6.5 |
| P/Ca+Phy+Coc+ | 3.7 | 0.2 | 0.5 | 7.1 | 2.8 | n.d. | 1.2 | 13.4 | |  | 1.6 | 6.9 |
| P/Ca-Phy-Coc±^2^ | 0.3 | <LOQ | 0.3 | n.d. | 1.2 | 0.5 | 0.4 | 23.8 | |  | 1.2 | 6.3 |
| pooled SEM | 0.21 | 0.03 | 0.08 | 0.60 | 0.34 | 0.03 | 0.13 | 1.66 | |  | 0.19 | 0.27 |
|  | *P-*values | | | | | | | | | | |  |
| *P/Ca* | <0.001 | - | 0.022 | <0.001 | <0.001 | <0.001 | <0.001 | <0.001 | |  | <0.001 | 0.004 |
| *Phytase* | <0.001 | 0.560 | 0.334 | <0.001 | 0.856 | ^-^ | <0.001 | <0.001 | |  | <0.001 | 0.746 |
| *Coccidostat* | 0.049 | - | 0.851 | 0.302 | 0.989 | 0.466 | 0.902 | 0.238 | |  | 0.090 | <0.001 |
| *P/Ca×Phy* | - | - | - | - | 0.168 | ^-^ | <0.001 | <0.001 | |  | 0.013 | 0.559 |
| *P/Ca×Coc* | - | - | 0.679 | 0.343 | 0.944 | 0.466 | 0.838 | 0.957 | |  | 0.766 | 0.660 |
| *Phy×Coc* | - | - | 0.598 | 0.313 | 0.900 | ^-^ | 0.541 | 0.997 | |  | 0.401 | 0.536 |
| *P/Ca×Phy×Coc* | ^-^ | - | - | ^-^ | 0.967 | ^-^ | 0.596 | 0.747 | |  | 0.620 | 0.337 |

^1^ Not shown isomers were not detectable (n.d.) or not quantifiable (<LOQ) in the majority of samples; n=7 pens

^2^Additional treatment, was not part of the three-factorial analysis

^3^ At least one of the following isomers: Ins(1,2,6)P_3_, Ins(1,4,5)P_3_, Ins(2,4,5)P_3_

Table S3 Effect of the experimental diets and the sampling section on the microbial composition (PERMANOVA analysis)

1. Treatments of the 2 x 2 x 2 factorial arrangement of treatments

|  | Crop and Ileum | Crop | Ileum |
| --- | --- | --- | --- |
|  | *P*-values | | |
| *Treatment* | 0.001 | <0.001 | <0.001 |
| *Section* | 0.001 |  |  |
| *Treatment x Section* | 0.969 |  |  |
|  |  |  |  |
| *P/Ca* | 0.055 | 0.195 | 0.201 |
| *Phytase* | 0.021 | 0.032 | 0.321 |
| *Coccidostat* | <0.001 | <0.001 | <0.001 |
| *P/Ca×Phy* | 0.147 | 0.068 | 0.812 |
| *P/Ca×Coc* | 0.001 | 0.008 | 0.007 |
| *Phy×Coc* | 0.009 | 0.105 | 0.070 |
| *P/Ca×Phy×Coc* | 0.025 | 0.378 | 0.044 |

1. Pairwise comparisons between treatments P/Ca-Phy-Coc-, P/Ca-Phy-Coc+, and P/Ca-Phy-Coc+

|  | Crop | Ileum |
| --- | --- | --- |
| P/Ca-Phy-Coc- vs P/Ca-Phy-Coc+ | 0.001 | <0.001 |
| P/Ca-Phy-Coc- vs P/Ca-Phy-Coc+ | 0.246 | 0.213 |
| P/Ca-Phy-Coc+ vs P/Ca-Phy-Coc+ | 0.003 | 0.001 |

Table S4 Effect of different coccidiostat treatments on performance traits of broilers

|  | Phase 2 (d10-24/25, n=7 pens) | | |  | Phase 1+2 (d1-24/25, n=7 pens) | | |  | d24/25 |
| --- | --- | --- | --- | --- | --- | --- | --- | --- | --- |
|  | ADG | ADFI | G:F |  | ADG | ADFI | G:F |  | BW |
|  | g/d | g/d | g/g |  | g/d | g/d | g/g |  | g |
| P/Ca-Phy-Coc- | 35^b^ | 50^b^ | 0.71 |  | 28^b^ | 37^b^ | 0.76 |  | 703^b^ |
| P/Ca-Phy-Coc+ | 40^a^ | 56^a^ | 0.72 |  | 32^a^ | 41^a^ | 0.77 |  | 785^a^ |
| P/Ca-Phy-Coc+/- | 37^ab^ | 52^ab^ | 0.71 |  | 30^ab^ | 39^ab^ | 0.77 |  | 752^ab^ |

^a–f^ Means within a column not showing a common superscript differ (*P* ≤ 0.05)

Table S5 Effect of different coccidiostat treatments on precaecal nutrient digestibility, InsP_6_ disappearance^1^ and foot ash^2^

|  | P digestibility | Ca digestibility | CP digestibility | Crop InsP_6_ disappearance | Ileum InsP_6_ disappearance | *My*o-Inositol | Foot ash | Foot ash |
| --- | --- | --- | --- | --- | --- | --- | --- | --- |
|  | % | % | % | % | % | g/kg DM | mg | % of DM |
| P/Ca-Phy-Coc- | 47.1 | 64.0 | 77.6 | -1.7^b^ | 47.7 | 1.4^ab^ | 568^b^ | 9.4^b^ |
| P/Ca-Phy-Coc+ | 48.0 | 61.1 | 77.5 | 4.5^a^ | 48.6 | 1.6^a^ | 639^a^ | 9.9^a^ |
| P/Ca-Phy-Coc+/- | 47.3 | 63.1 | 76.5 | -0.1^b^ | 50.3 | 1.2^b^ | 606^b^ | 9.5^b^ |

^1^ n=7 pens

^2^ n=70 birds

^a–f^ Means within a column not showing a common superscript differ (*P* ≤ 0.05)

Table S6 Effect of different coccidiostat treatments on blood metabolites and pH in digesta

|  | P_i_  mmol/l | Ca  mmol/l | ALP  U/l | *Myo*-Inositol  µmol/ml | Crop pH | Ileum pH |
| --- | --- | --- | --- | --- | --- | --- |
| P/Ca-Phy-Coc- | 1.2^ab^ | 3.1^ab^ | 9239^a^ | 0.33 | 5.1 | 6.2 |
| P/Ca-Phy-Coc+ | 1.4^a^ | 3.3^a^ | 6525^b^ | 0.31 | 5.0 | 6.6 |
| P/Ca-Phy-Coc+/- | 1.2^b^ | 2.9^b^ | 8457^ab^ | 0.30 | 5.2 | 6.3 |

^1^ P_i_: inorganic phosphate, Ca: calcium and ALP: alkaline phosphatase in blood serum; *myo*-inositol in blood plasma; n=14 birds; pH values: n=7 pens

^a–f^ Means within a column not showing a common superscript differ (*P* ≤ 0.05)


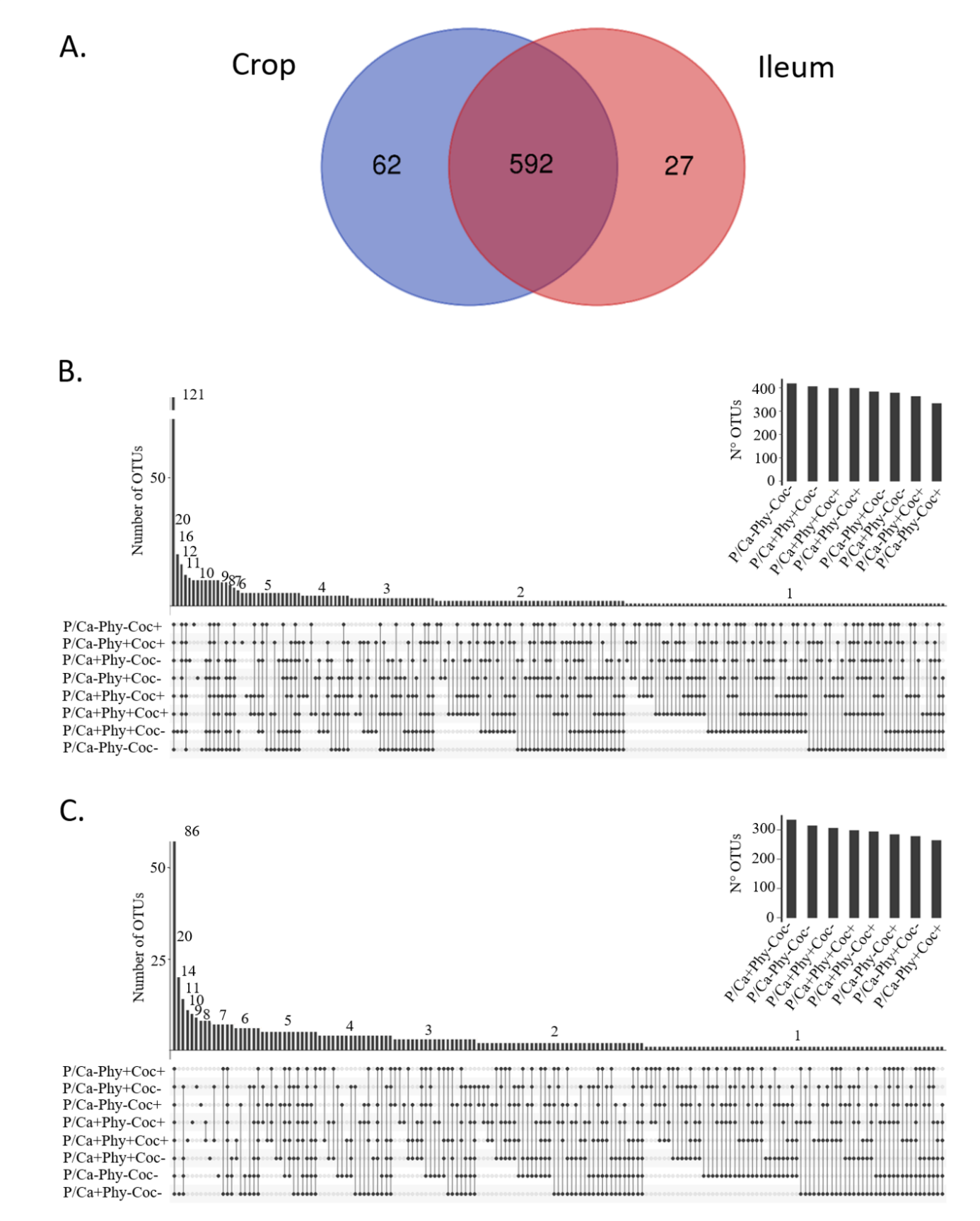


Figure S1 Distribution of the Operational Taxonomic Units (OTUs). Venn diagram showing the intersection between the crop and ileum (A). Matrix layout to show the number of OTUs for the core microbiota at Operational Taxonomic Units level found in the crop (B) and ileum (C)





Figure S2 Cluster analysis for crop (A) and ileum (B) digesta samples





Figure S3 Non-metric multi-dimensional scaling plot illustrating the global bacterial community structure. Dietary treatments are shown based on their replicates obtained for the digesta samples of crop (A) and ileum (B). The symbols represent one pooled sample from each pen comprising all Operational Taxonomic Units


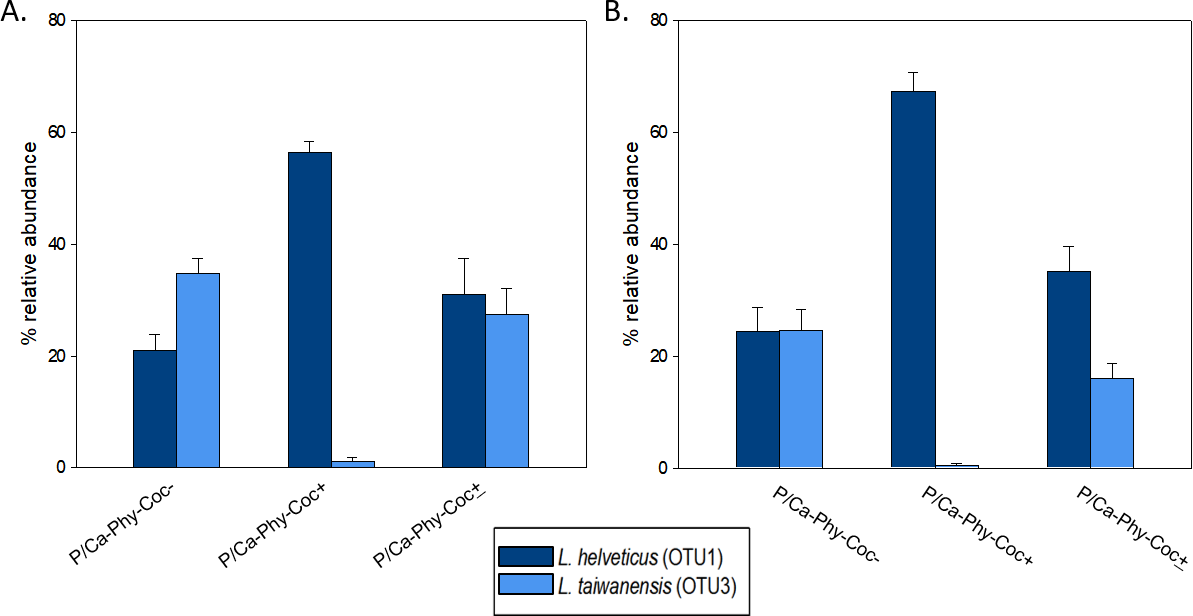


Figure S4 Relative abundance for more abundant Operational Taxonomic Units (OTUs) in Crop (A) and ileum (B) digesta samples
